# Long-term multidisciplinary treatment including proton therapy for a recurrent low-grade endometrial stromal sarcoma and pathologically prominent epithelial differentiation: an autopsy case report

**DOI:** 10.1186/s12905-020-01019-0

**Published:** 2020-07-25

**Authors:** Osamu Maeda, Tetsuro Nagasaka, Makoto Ito, Tomoyo Mitsuishi, Fumihiko Murakami, Toshio Uematsu, Yukiko Hattori, Hiromitsu Iwata, Hiroyuki Ogino

**Affiliations:** 1 Department of Gynecology, Meijo Hospital, Sannomaru 1-3-1, Naka-ku, Nagoya, 460-0001 Japan; 2 Department of Medical Technology, Nagoya University, School of Health Science, Nagoya, Japan; 3 Department of Pathology and Laboratory Medicine, Kariya-Toyota General Hospital, Kariya, Aichi Japan; 4 Department of Pathology and Laboratory Medicine, Meijo Hospital, Nagoya, Japan; 5 Department of Thoracic Surgery, Meijo Hospital, Nagoya, Japan; 6 Department of Surgery, Meijo Hospital, Nagoya, Japan; 7 Department of Radiation Oncology, Nagoya Proton Therapy Center, Nagoya City West Medical Center, Nagoya, Japan

**Keywords:** Adenocarcinoma, Autopsy, Chemotherapy, Hormonal therapy, Low-grade endometrial stromal sarcoma, Mesenchymal epithelial-like differentiation, Neuroendocrine tumor, Pazopanib, Proton therapy

## Abstract

**Background:**

Long-term follow-up reports of low-grade endometrial stromal sarcoma (LGESS) including its clinical course and pathological data are rare. We previously reported the case of a Japanese woman diagnosed with LGESS, who was treated with multidisciplinary therapy. She had been suffering from uterine cervical tumor diagnosed as cervical polyps, or fibroid *in statu nascendi,* since 24 years old. The patient had survived for 25 years with the disease. This report presents her progress and pathological change since the previous report.

**Case presentation:**

At age 45, the patient experienced a relapse of the remnant LGESS tumor between the right diaphragm and liver. Although chemotherapy was not effective, the tumor was eliminated by proton therapy. At age 46 years, the remnant tumors outside the irradiated field were resected. The disease was originally diagnosed as “neuroendocrine carcinoma (NEC)” using the surgical specimen. Therefore, cisplatin and irinotecan combination chemotherapy were administered to treat the remnant dissemination. After 4 cycles of chemotherapy, the liver metastases had enlarged and were resected surgically. Consequently, no remnant tumor was visible in the abdominal cavity at the end of the surgery. To determine the origin of NEC, we examined the previously resected specimens obtained from her ileum at age 40 years. A boundary between the LGESS and neuroendocrine tumor grade 2 (NET G2)-like lesion was found in the tumor, indicating that the origin of these tumors was LGESS. After less than 2 years of chemotherapy and undergoing surgery, a relapse of the tumor in the liver induced biliary duct obstruction with jaundice, which was treated with endoscopic retrograde biliary drainage. Although pazopanib prolonged her life for 10 months, she died from sepsis at age 49 years, which was caused by the infection that spread to the liver metastatic tumor via the stented biliary ducts. Autopsy revealed adenocarcinoma-like differentiation of the tumor.

**Conclusion:**

This LGESS patient has survived for a long time owing to multidisciplinary treatment including proton therapy. The LGESS tumor differentiated to NET G2-like tissue and then further to adenocarcinoma-like tissue during the long-term follow-up.

## Background

Low-grade endometrial stromal sarcoma (LGESS) is a rare condition [1, 2]. Post-relapse survival of patients with endometrial stromal sarcoma can be expected to be more than10 years [3]. Clinical trial results have recently been reported for other soft-tissue sarcomas, but not specifically for LGESS. Evidence suggests that eribulin [4] and pazopanib [5] are effective treatments for sarcomas, including those with orthopedic involvement. Newer therapies are being introduced into routine clinical treatment as well. In the pathological field, endometrial stromal sarcoma shows a varied morphological appearance, and clinicopathologic studies about endometrial stromal sarcoma has been described [6, 7]. However, it is difficult even for experienced pathologists to diagnose them correctly.

We had previously published a case on LGESS in 2015 [8]. A Japanese woman had been suffering from uterine cervical tumor diagnosed as cervical polyps, or fibroid *in statu nascendi*, at age 24. She underwent 10 resections in 10 years without any diagnosis of malignancy. She also had a child during this period at the age of 28. When she was 34 years old, she started experiencing from lower abdominal pain; and subsequently, we detected a 10-cm tumor behind her uterus. Laparotomy was the preferred option due to the advanced stage and malignant nature of the ovarian tumor. However, we later discovered that the tumor was in fact advanced LGESS. Although she had a fair number of tumor remnants in the abdominal cavity after her surgery, a combination of gemcitabine and docetaxel chemotherapy (GD) proved effective, and the tumor remnants disappeared completely. The specimens previously resected trans-vaginally were reviewed pathologically. Indeed, the lesions included LGESS elements. Therefore, this disease was proved to have started since she was 24 years old. However, at age 40, a recurrent tumor was detected in the pelvic cavity and was resected soon after. At age 42, the LGESS recurred around the right diaphragm and the liver. Because GD proved ineffective, medroxyprogesterone (MPA), leuprorelin, and anastrozole were added one by one, and paclitaxel, and carboplatin combination chemotherapy (TC) was added after pleural effusion and ascites had decreased. The patient recovered and survived after administering the five drugs together.

Long-term, detailed reports of LGESS patients with multidisciplinary treatments and histological investigation are rare. Herein, we present details of the patient’s clinical course after our previously published report in 2015, and describe the histological changes of the LGESS tumor after a long-term multidisciplinary treatment by investigating the patient’s surgical and autopsy specimens.

## Case presentation

**Fig. 1:**
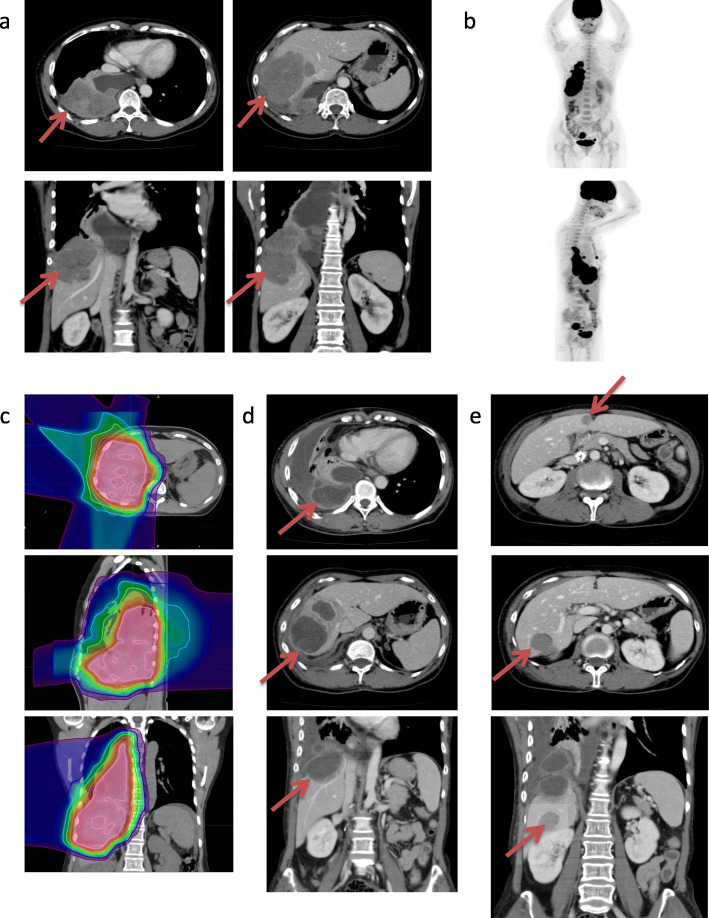
Diagnostic image after recurrence around the right diaphragm, liver, and thoracic cavity. **a** Enhanced computed tomography (CT) image before proton therapy, and after chemotherapy. Red arrows indicate the tumor between the right diaphragm and liver. **b** Positron emission tomography-CT image during the same time as in “a”. **c** Planning of proton therapy. **d** Enhanced CT after 4 months of proton therapy. The tumors in the irradiation field disappeared and were liquefied. Red arrows indicate the liquefied tumors. **e** Enhanced CT image when liver metastases increased. Red arrows indicate the liver metastases

**Fig. 2:**
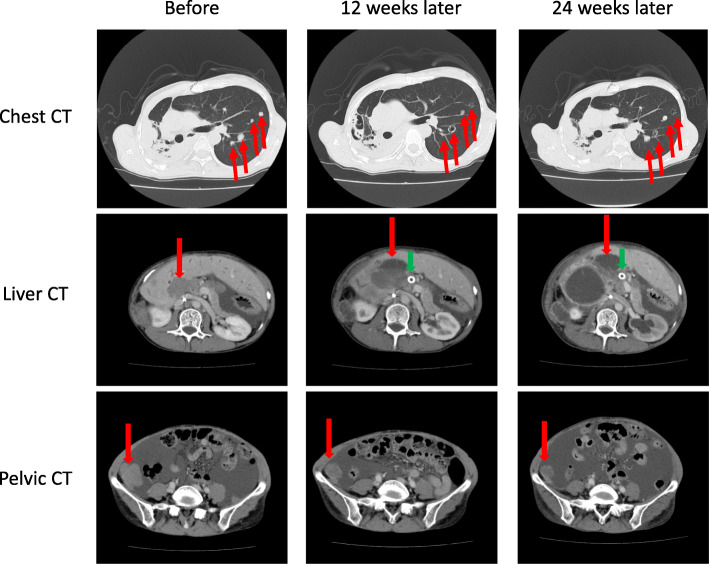
Computed tomography (CT) images showing the effect of pazopanib (800 mg/day). The upper, middle, and lower lines indicate chest CT, liver CT, and pelvic CT, respectively. The left, central, and right rows indicate before administration of pazopanib, 12 weeks later, and 24 weeks later, respectively. Red arrows and green arrows indicate tumors, and common bile duct stents, respectively. Chest CT images show that lung metastases reduced after 12 weeks of treatment and recurred after 24 weeks, in comparison with the first scan. Liver CT images show that liver metastatic tumors reduced and liquefied, while continuing the administration of pazopanib. Pelvic CT images show that the right pelvic dissemination tumor reduced, and there was a decrease in ascites after 12 weeks of treatment and an increase in ascites 24 weeks later

**Fig. 3:**
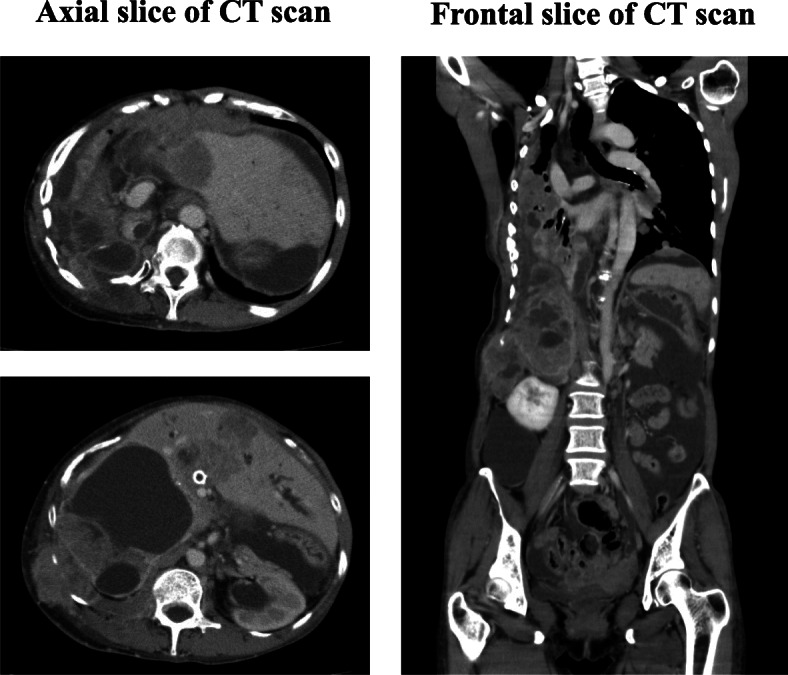
Diagnostic computed tomography (CT) image before the patient’s death. Axial slice and frontal slice CT scans at 4 weeks before the patient’s death. The tumor invaded into the subcutaneous tissue via the intercostal muscle. Furthermore, the tumor invaded into the pleural cavity from the liver surface via the right diaphragm

Case history: We have shown above a background of this case. During the course of LGESS progression, the patient had one gravida and one caesarian section. At the time of diagnosis, she had no relevant past history or family history. When she was 45 years old, a remnant tumor between her right diaphragm and liver relapsed. TC (Table 1a: paclitaxel (140 mg/m^2^) and carboplatin (area under the curve (AUC): 4), 7 cycles), and ifosfamide and doxorubicin combination chemotherapy (Table 1b: ifosfamide (2 g) for 4 days + doxorubicin (30 mg) for 2 days, 3 cycles), in addition to hormonal therapy, did not decrease the tumor size (Fig. 1a and b). At age 46, the tumor was treated with proton therapy 70.2 GyE/26 Fr, for 40 days (Table 1c, Fig. 1c). The tumors in the irradiation field almost disappeared and liquefied (Fig. 1d). The remnant tumors in the thoracic and abdominal cavity, which were out of the irradiation field, were resected (Table 1d). Originally, the tumor was pathologically diagnosed as “neuroendocrine carcinoma (NEC).” Thrombosis in the inferior vena cava led to the discontinuation of MPA (Table 1e). Thereafter, 4 cycles of cisplatin and irinotecan combination chemotherapy were administrated for NEC (Table 1f: cisplatin 70 mg + irinotecan 70 mg, 4 cycles, every 4 weeks). Seven months after the previous surgery, two enlarged liver metastases (Table 1g, Fig. 1e) were detected, and another surgery was performed. After the resection of the two liver metastases and a small number of disseminated tumors, no disseminated tumors remained in the abdominal cavity. The pathological diagnosis of the resected tumor was also NEC. Although the chemotherapy was not effective for the larger liver metastases, it effectively eliminated smaller disseminated tumors. Therefore, 2 cycles of cisplatin and irinotecan combination chemotherapy were administered at 1 month and at 4 months after the operation to prevent the recurrence of dissemination (Table 1h: cisplatin 70 mg + irinotecan 70 mg, 2 cycles, every 3 months). However, chemotherapy was discontinued because of diarrhea, lack of appetite, and general fatigue. Following this, TC (Table 1i: paclitaxel (140 mg/m^2^) and carboplatin (AUC: 4), 1 cycle, Table 1l: paclitaxel (140 mg/m^2^) and carboplatin (AUC: 4), 3 cycles), eribulin (Table 1k: eribulin 1.4 mg/m^2^, 3 cycles.), and paclitaxel single agent (Table 1m: paclitaxel (140 mg/m^2^) 2 cycles) were administered. However, these chemotherapies did not show any efficacy. Surgery for liver metastases and dissemination in the spleen (Table 1j) were successful in controlling the disease. At age 48, common biliary duct obstruction by enlarged liver metastatic tumor induced jaundice and liver dysfunction (Table 1n). Common bile duct stents were inserted using endoscopic retrograde biliary drainage. After recovery from the condition, pazopanib (800 mg/day) was started with leuprorelin, and anastrozole (Table 1o). Computed tomography (CT) images are shown in Fig. 2. Before administration, multiple lung metastases, a large hepatic hilar metastatic tumor, large volume of ascites, and pelvic cavity disseminations were detected. After 12 weeks of administration, the medicine was effective. The sizes of tumors invading the right lung were reduced. The left lung metastases and pelvic disseminations reduced, and the liver metastasis partially resolved too. However, after 24 weeks of administration, the lung metastases markedly increased along with the ascites. After 3 months of the best supportive care, she had a fever and developed a hard-raised palpable lesion under the skin on the right lower back of the chest. Blood culture revealed bacteremia due to *Enterobacter cloacae*. CT images at 4 weeks before her death are shown in Fig. 3. The tumor between the right diaphragm and the liver invaded into the right lung via the right thoracic cavity and into the subcutaneous tissues via the intercostal muscles. Cystic lesions were observed in the liver, which were shown in the axial slice. Finally, she died at age 49 due to sepsis after long-term treatment of advanced refractory LGESS (Table 1p). Autopsy was then performed. The infection progressed to the liver metastatic tumor via the stented biliary ducts.

**Table 1:** The course of treatment for this Low-grade Endometrial Stromal Sarcoma case

Age	The patient’s condition	Treatment	The outcome
*a*: 45 y 1 m	The tumor between liver and right diaphragm increased	TC^a^, 7 cycles	SD
*b*: 45 y 9 m	The tumor between liver and right diaphragm increased	Ifosfamide + Doxorubicin^b^, 3 cycles	PD
*c*: 45 y 11 m	The tumor between liver and right diaphragm increased	Proton therapy, 70.2 GyE/26 Fr. For 40 days	PR (Fig. 1)
*d*: 46 y 3 m	The tumors outside of the irradiation field remained	Surgery for the left pleural and abdominal tumors	Neuroendocrine carcinoma
*e*: 46 y 5 m	Thrombosis in inferior vena cava distal part	An inferior vena cava filter. Discontinued MPA^c^.	Disappeared 11 months later.
*f*: 46 y 5 m	Small disseminations remained at the end of the surgery	^d^Cisplatin + Irinotecan, 4 cycles, every 4 weeks	PD
*g*: 46 y 8 m	Liver metastasis increased	Surgery for liver metastasis.	No remnant in surgical field
*h*: 46 y 11 m	Effective for small dissemination tumors	^d^Cisplatin + Irinotecan, 2 cycles, every 3 months	Discontinued by dehydration
*i*: 47 y 4 m	Multiple lung metastases recurred	TC^a^, one cycle.	Discontinued by pneumothorax
*j*: 47 y 11 m	Abdominal and spleen dissemination increased	Splenectomy and dissemination resection	No remnant in surgical field
*k*: 48 y 1 m	Vaginal bleeding by recurrence in vaginal stump	Eribulin 1.4 mg/m^2^, 3 cycles.	PD
*l*: 48 y 4 m	The tumors increased.	TC^a^, 3 cycles	Hypersensitivity for carboplatin
*m*: 48 y 7 m	Discontinuation of carboplatin	Paclitaxel (140 mg/m^2^) 2 cycles	PD
*n*: 48 y 8 m	Jaundice and liver dysfunction	The common bile duct stents (Fig. 2).	Recovered liver dysfunction
*o*: 48 y 9 m	Liver metastasis increased	Pazopanib hydrochloride 800 mg/day started	SD to PD (Fig. 2)
*p*: 49 y 7 m	Bacterial infection from liver tumor via bile duct	Antibiotics and palliative care	Died with sepsis (Fig. 3)

Alphabet letters before age indicate sentences concerning events in the main text. ^a^TC: paclitaxel (140 mg/m^2^) and carboplatin (area under the curve (AUC): 4) combination chemotherapy. ^b^Ifosfamide + Doxorubicin: Ifosfamide (2 g) for 4 days + Doxorubicin (30 mg) for 2 days. ^c^MPA: Medroxyprogesterone 600 mg/day. Hormonal therapy by MPA, leuprorelin 3.75 mg every 28 days, and anastrozole 1 mg/day was continued until thrombosis (*e*) except for the periods of proton therapy and surgery. After this time, hormonal therapy by leuprorelin, and anastrozole was continued until patient died. ^d^Cisplatin + Irinotecan: Cisplatin 70 mg + Irinotecan 70 mg. *PR* partial response, *SD* stable disease, *PD* progress disease

**Fig. 4:**
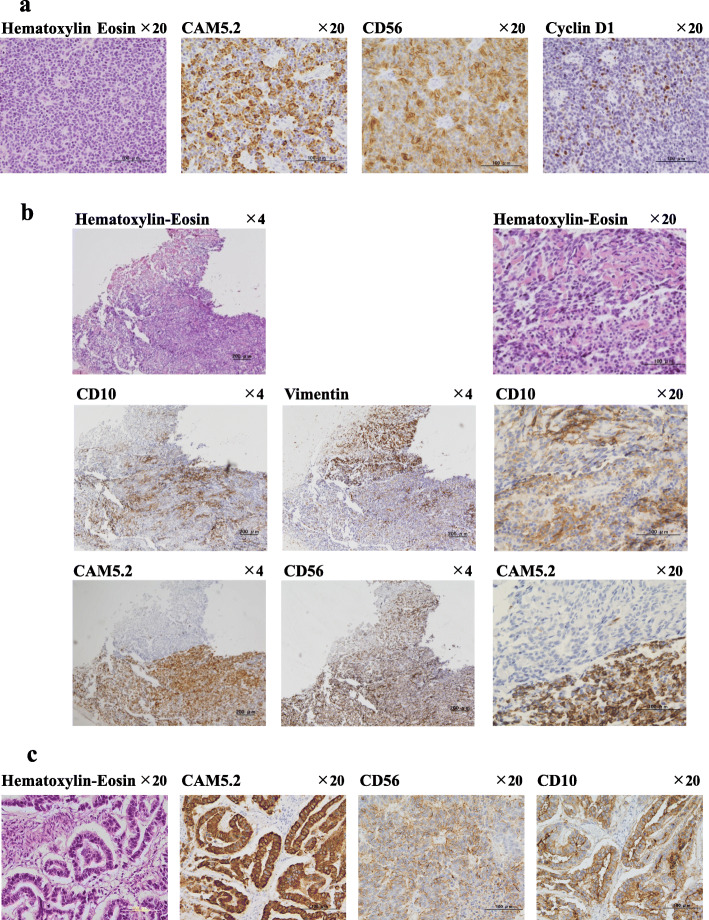
Microscopic images of hematoxylin-eosin stain and immunohistochemical staining of the tumors. **a** Tumors resected in the abdominal cavity at the age of 46 years. The targeted antigens included CAM5.2, CD56, and Cyclin D1. The tumor cells exhibited a rosette-form or ribbon-shaped feature during hematoxylin-eosin staining. Immunohistochemical analysis revealed that the tumor cells were strongly positive for CAM5.2 and CD56, but weakly positive for cyclin D1 expression. **b** The metastatic tumor resected from the ileum of the patient at the age of 40 years. Hematoxylin-eosin staining and CD10, vimentin, CAM5.2, and CD56 immunohistochemical staining showed that the LGESS and NET G2-like sections were observed in the upper and lower portions, respectively. **c** The autopsy specimens show glandular-shaped characteristics during hematoxylin-eosin staining. The tumor cells were strongly positive for CAM5.2 and CD10 and moderately positive for CD56

Pathological review: The materials and methods of immunohistochemistry are described in Table 2. The tumors disseminated in the thoracic and abdominal cavities were initially diagnosed as NEC at 46 years of age (Fig. 4a and Table 3b). The tumor cells exhibited a rosette form or a ribbon-shaped feature during hematoxylin-eosin staining. Immunohistochemical analysis revealed that the tumor cells were strongly positive for CAM5.2 and CD56 and were weakly positive for chromogranin A, a marker for epithelial or neuroendocrine cells. Mitoses were examined in 15/10 high power fields, and the Ki-67-positive ratio was 15%. Although these findings indicate neuroendocrine tumor grade 2 (NET G2) according to the World Health Organization criteria [9], we diagnosed the tumor as NEC because of the numerous metastases. Abdominal and thoracic dissemination, invasion of the surrounding tissue, and penetration of the diaphragm were present, which were suggestive of NEC rather than of NET G2. Additional immunohistochemical staining was performed for comparison with LGESS. The expression of CD10 and vimentin by tumor cells was weakly and moderately positive, respectively. The expression of CD99, inhibin α, estrogen receptor, and progesterone receptor was negative in the tumors, whereas cyclin D1 expression was weakly positive. These observations indicate that these tumors mainly had epithelial characteristics and partly preserved LGESS characteristics.

**Table 2:** Antibodies used for characterization of the Tumors

Antibody	Source
anti-cytokeratin (clone: CAM5.2, mouse)	Becton, Dickinson and Company BD Biosciences, San Jose, CA, USA
anti-CD56 monoclonal (clone: 1B6, mouse)	Nichirei Bioscience Co. Ltd. Tokyo, Japan
anti-chromogranin A polyclonal (rabbit)	Nichirei
anti-human Ki-67 monoclonal (clone MIB-1, mouse)	Agilent Technologies, Santa Clara, CA, USA
anti-CD10 monoclonal (clone: 56C6, mouse)	Nichirei
anti-vimentin monoclonal (clone V9, mouse)	Nichirei
anti-estrogen receptor α monoclonal (clone EP-1, rabbit)	Agilen
anti-progesterone receptor monoclonal (clone PgR636, mouse)	Agilent
anti-CD99 monoclonal (clone O13, mouse)	F.Hoffmann-La Roche Ltd., Basel Switzerland
anti-inhibin α monoclonal (clone R1, mouse)	Agilent
anti-cyclin D1 monoclonal (clone SP4-R, rabbit)	Ventana Medical Systems, inc. Tuscon, AZ, USA

Methods of immunohistochemistry: Tissue sections were cut to 4 μm thick and immunohistochemically stained using paraffin sections of surgical specimens. Heat-induced epitope retrieval was performed by heating deparaffinized sections in buffer (Nichirei Histofine, pH 9.0) (Nichirei Bioscience Co. Ltd. Tokyo, Japan) for 30 min at 98 °C for CAM5.2, CD56, Ki-67, CD10, estrogen receptor, progesterone receptor, CD99, inhibin α, and cycline D1. The slides were developed using 3,3′-Diaminobenzidine and were counterstained with hematoxylin. All antibodies were used at a dilution of 1:50

**Table 3:** Comparison of antigen expression in surgical specimens by immunochemical staining

Antigens	CD10	Vimentin	Estrogen receptor	Progesterone receptor	CAM5.2	CD56	Chromogranin A	Ki-67 Positive ratio Mitosis
a: Ileal recurrent tumor at 40 years of age (Fig. 4b)								
The LGESS section	1+	3+	0	0	0	2+	0	3% < 1/10 HPF
The NET-like section	2+	1+	0	0	3+	3+	0	5% 5/10 HPF
b: Disseminated tumor at 46 years of age (Fig. 4a)								
	1+	2+	0	0	3+	3+	1+	15% 15/10 HPF
c: Autopsy at 49 years of age (Fig. 4c)								
Adenocarcinoma-like section	3+	ND	ND	ND	3+	2+	ND	ND ND

0, negative; 1+, weakly positive; 2+, moderately positive; 3+, strongly positive

Microscopic photographs of staining for CD10, vimentin, CAM5.2, CD56, and cyclin D1 are shown in Fig. 4. *HPF* high power field, *ND* not done

To determine the origin of NEC, we examined the previously resected specimen. There was no NEC in the uterus, in either the adnexa or disseminated tumors, when the first laparotomy was performed at the age of 34 years. The recurrent tumor resected from the ileum at the age of 40 years, which was diagnosed as LGESS, was investigated retrospectively (Fig. 4b and Table 3a). Examination of the details showed that there were small “NET G2-like” lesions in the specimen and at the boundary between the LGESS and NET G2-like lesion. The LGESS and NET G2-like sections were observed in the upper and lower portions, respectively. At 4× magnification of hematoxylin-eosin-stained specimens, the cell density was lower in the LGESS section than in the NET G2-like section. At 20× magnification, the LGESS section was seen to have hyalinization and oval-to-short spindle cells with indistinct cytoplasm. In contrast, cells in the NET G2-like section of the same specimen had ribbon-shaped characteristics and exhibited a rosette formation. Immunohistochemical analysis showed that CD10 expression was weakly positive in the LGESS section and moderately positive in the NET G2-like section. Vimentin expression was strongly positive in the LGESS section but weakly positive in the NET G2-like section. Although CD10 and vimentin indicate stromal tissues, the strength of the staining was reversed. Estrogen and progesterone receptors were negative in both sections. CAM5.2 expression was negative in the LGESS section but strongly positive in the NET G2-like section. The border between these sections was very clear. CD56 expression was moderately positive in the LGESS section and strongly positive in the NET G2-like section. The staining strength showed gradation from the LGESS section to the NET G2-like section. Chromogranin A expression was negative in both the LGESS and NET G2-like sections. In the LGESS and NET G2-like sections, the Ki-67 positive ratio was 3 and 5%, and mitoses were observed in < 1/10 and 5/10 high power fields, respectively. The malignant characteristics were greater in the NET-like section than in the LGESS section. Mixed mesenchymal and epithelial characteristics were detected in the lesion.

Autopsy findings showed that the NET did not originate from the lung, digestive tract, pancreas, and other organs. Tumorous pleuritis was detected in the right thoracic cavity. Abdominal, colon serosal, and pelvic disseminations were observed. Multiple nodular metastatic tumors were detected in the liver. The metastatic tumor with a maximum size of 10-cm in the liver included a necrotic and a pus-filled cystic lesion. It was concluded that the cause of death was sepsis caused by the infection that spread to the liver metastatic tumor via the stented biliary duct. Microscopically, the metastatic tumor in the right lung had glandular-shaped characteristics and exhibited an epithelial transformation, which showed adenocarcinoma-like characteristics with a glandular formation (Fig. 4c and Table 3c). Immunohistochemical analysis revealed that the tumor cells were strongly positive for CAM5.2 and CD10 and moderately positive for CD56. The tumor cells had both epithelial and mesenchymal characteristics.

## Discussion and conclusions

Proton therapy was very effective for recurrent and refractory LGESS. The patient survived for a long period with a series of multidisciplinary treatment. Moreover, this case report is the first observation of the two-stage differentiation of LGESS into NET G2-like tissues and further into adenocarcinoma-like tissues.

### Proton therapy

We believe that this case report is the first to describe a very effective use of proton therapy for LGESS. This extremely large tumor, which was resistant to multiple anticancer drugs, was very difficult and dangerous to resect with surgery. Conventional radiation therapy would not have had the same effect as proton therapy. Even in large tumor, the Bragg peak of protons can deliver high dose to the tumor while sparing the surrounding normal tissues. The energy of the protons is accumulated in the large tumor and hardly penetrate through the tumor to its opposite side. Therefore, any damage to nearby organs is minimal. This therapy resulted in substantial reduction of the tumor, which was in an unresectable location; thus, we considered it as the best treatment choice for this LGESS. We think that proton therapy is a viable option for treating very large and potentially unresectable LGESS.

### Surgery

The patient had undergone surgeries three times in this period, with a total of five surgeries throughout the course of this disease. The patient underwent her first surgery when she was 34 years old. Not only was it an effective debulking surgery, but it was found that the disease was advanced LGESS rather than a malignant ovarian tumor. The surgery was effective for prolonging her life despite her severe medical condition. Furthermore, the pathological diagnosis obtained by the surgery contributed to the discovery of minimal elements of LGESS by a retrospective study of transvaginal resected tumors. The second surgery at age 40 contributed to prolonging life by preventing intestinal obstruction and to retrospective pathological diagnosis of NET G2-like lesion. If we did not have the specimen, we could not find the origin of NET G2-like lesion differentiated LGESS. The third surgery (at age 46; Table 1d) was a tumor debulking procedure to maintain left lung and thorax function, and to prevent intestinal obstruction. The pathological diagnoses of this surgery were NEC, which indicated change of malignant tumor. The fourth and fifth surgeries (Table 1g, j) were also tumor debulking procedures. As mentioned above, the repeated surgeries had effectively prolonged the patient’s life, and the surgical specimens served as change indicators of condition’s severity.

### Medications

At age 34, this patient was diagnosed as having LGESS. At that time, the patient was treated with surgery and repeated GD. A total of 20 cycles of GD were administered. The last 3 cycles of GD were not effective, as the tumor recurred around the right diaphragm and liver at age 42. Then, the tumor was treated with MPA, leuprorelin, anastrozole, and TC. TC was totally administered for 29 cycles. In the first half of TC administration, the chemotherapy was very effective, and the tumor showed marked reduction. Conversely, in the latter half of TC administration, the tumor became chemo-resistant and the effect was limited. After diagnosis of NEC, cisplatin and irinotecan combination chemotherapy was administered. Although the chemotherapy was ineffective for larger tumors of the liver metastases, it effectively treated the small disseminated tumors, as indicated by our later surgical findings (Table 1g). In total, 6 cycles of the chemotherapy were administered, but this regimen was not repeated continuously because of the side effects, such as general fatigue, dehydration, and loss of appetite. TC caused more tolerable side effects for the patient than cisplatin and irinotecan combination chemotherapy. Ifosfamide and doxorubicin combination chemotherapy, and eribulin single agent were ineffective for this tumor. In retrospect, we believe that TC had an effect to delay the growth of the tumor, even in the latter half of TC administration. Therefore, we thought that TC was the chemotherapy regimen of last resort for this case after acquisition of GD resistance. TC with hormonal therapy contributed to a lack of disease progression against chemo-resistance.

The hormonal therapy consisting MPA, leuprorelin, and anastrozole had been continued for 6.5 years. We believe that the side effects were thoracic bleeding from LGESS tumor by Flare-up at the beginning of leuprorelin administration [8] and thrombosis in the inferior vena cava by MPA after surgery, or repeated dexamethasone administration with TC. These side effects would have been lethal if appropriate treatment had not been undertaken. Other side effects were not observed in the long term. We believe that hormonal therapy contributed to enhance cytotoxicity of TC by controlling LGESS tumor activity, because the patient had controlled ascites and pleural effusion in the period before administering TC. We think that hormonal therapy is also a viable option for treating very large and potentially unresectable LGESSs. We could not discontinue the hormonal therapy after original diagnosis of the “NEC,” although the hormonal therapy did not have an effect on general NEC. The “NEC” or the “NET G2-like lesion” did not express estrogen receptor or progesterone receptor. We believed that previously existing LGESS was controlled by the hormonal therapy, and the patient relied on the hormonal therapy.

We also believe that pazopanib played a major role in increasing her life expectancy in the terminal stage. Although the tumor regressed temporally with pazopanib treatment, the effect did not last. Because this LGESS acquired chemo-resistance from repeated exposure to chemotherapy and spread systemically, it was very difficult to treat. We think that pazopanib remains an effective treatment option for refractory LGESS.

### Pathological differentiation

At age 34, our patient was diagnosed as having LGESS. At age 40, the recurrent tumor was diagnosed as LGESS. However, retrospective re-examination revealed that the specimen included a small NET G2-like lesion. When the tumor recurred at 42 years of age, the small specimen obtained via needle biopsy was diagnosed as LGESS. Therefore, the main component of the tumor was LGESS until the patient was 42 years old. At 46 age, the tumor was diagnosed as NEC. The specimens showed NET G2-like characteristics, and clinical findings showed multiple metastases. Moreover, autopsy specimens showed adenocarcinoma-like characteristics.

Generally, NET originated from the cells of the endocrine or nervous system. The diffuse neuroendocrine system distributes throughout the whole body. Therefore, these tumors can appear in any organ. NET often originates from the lung, digestive tract, and pancreas. In gynecology, reports of these tumors are rare, with a small amount of available information published in one review [10]. The CT findings in this case revealed no new origin of NET at the same time; thus, we suspected that the original tumor was LGESS. Therefore, we re-examined the original specimen when the patient was 40 years old. We found a small region of NET G2-like tissue within the LGESS. The specimen had not been exposed to the hormonal therapy, because the hormonal therapy started from 42 years of age. Therefore, the hormonal therapy did not affect the generation of the NET G2-like lesion. We considered that this NET G2-like lesion grew, and the LGESS lesion was eliminated through the hormonal and cytotoxic chemotherapy [8]. The NET G2-like lesion had a different hormonal response because it did not express estrogen or progesterone receptors. Both hormone receptors were positive in the specimen taken when the patient was 34 years old [8]. We believe that this difference in hormonal response affected the chemosensitivity and growth. Therefore, the NET G2-like lesion remained and grew. Endometrial stromal sarcoma has been shown to express cytokeratin expression and to demonstrate glandular/epithelial differentiation [11, 12]. A uterine tumor resembling an ovarian sex cord tumor is reported to have undergone focal epithelial-like differentiation [13, 14]. These changes in characteristics make the differential diagnosis difficult. In this case, all resected tumors from the operations at age 46 (Table 1d, g) showed NET G2-like findings, which made the differential diagnosis very difficult. If no information about the previously resected tumor at 40 years of age had been available, we could not have made a diagnosis of LGESS with NET G2-like differentiation.

Autopsy findings showed that NET G2-like lesion did not originate from the thoracic or abdominal organs, which confirmed that LGESS is the origin of NET G2-like tumor. This malignant tumor showed many disseminated tumors, nodular metastatic tumors, and direct invasion of the tumor between the liver and right diaphragm to the thoracic cavity and to the right lung via the diaphragm, as well as invasion to the subcutaneous tissue via the intercostal muscle. The characteristics are different from those of LGESS. Long-term multidisciplinary treatment including chemotherapy and hormonal therapy would change the characteristics of LGESS. Microscopic findings of autopsy specimens showed adenocarcinoma-like characteristics, which showed glandular tissue-like shaped tissues. This indicated that LGESS differentiated further to epithelial-like tissues. These findings would greatly help in diagnosing LGESS with differentiation after long-term multidisciplinary treatment.

Finally, the advanced LGESS recurred repeatedly, and the malignancy progressed over time. The LGESS in our case differentiated into NET G2-like lesions and further into adenocarcinoma-like lesions during the long-term follow-up. We considered that this change showed the differentiation of the LGESS tumor from mesenchymal to epithelial-like lesions. Remarkably, the patient survived until 49 years of age suffering the disease for 25 years, and had a son while having the disease (20-year old at the time of her death). However, multidisciplinary treatments with the introduction of new therapies resulted in long-term survival. This report will give hope to patients with refractory LGESS.

## Data Availability

The datasets used and/or analysed during the current study are available from the corresponding author on reasonable request.

## Abbreviations

AUC: Area under the curve
CAM: Cell adhesion molecule
CD: Cluster of differentiation
CT: Computed tomography
G2: Grade 2
GD: Gemcitabine and docetaxel combination chemotherapy
HPF: High power field
LGESS: Low-grade endometrial stromal sarcoma
MPA: Medroxyprogesterone acetate
NEC: Neuroendocrine carcinoma
NET: Neuroendocrine tumor
PET-CT: Positron emission tomography-computed tomography
TC: Paclitaxel, and carboplatin combination chemotherapy
